# Transcriptome Pyrosequencing of the Parasitoid Wasp *Cotesia vestalis*: Genes Involved in the Antennal Odorant-Sensory System

**DOI:** 10.1371/journal.pone.0050664

**Published:** 2012-11-30

**Authors:** Osamu Nishimura, Carla Brillada, Shigenobu Yazawa, Massimo E. Maffei, Gen-ichiro Arimura

**Affiliations:** 1 Global COE Program: Evolution and Biodiversity, Graduate School of Science, Kyoto University, Kyoto, Japan; 2 Department of Life Sciences and Systems Biology, Plant Physiology Unit, Innovation Centre, University of Turin, Turin, Italy; 3 Center for Ecological Research, Kyoto University, Otsu, Japan; Duke University, United States of America

## Abstract

*Cotesia vestalis* is an endoparasitic wasp that attacks larvae of the diamondback moth (*Plutella xylostella*), a herbivore of cruciferous plants. Females of *C. vestalis* use herbivore-induced plant odorants released from plants infested by *P. xylostella* as a host-searching cue. Transcriptome pyrosequencing was used to identify genes in the antennae of *C. vestalis* adult females coding for odorant receptors (ORs) and odorant binding proteins (OBPs) involved in insect olfactory perception. Quantitative gene expression analyses showed that a few OR and OBP genes were expressed exclusively in the antenna of *C. vestalis* adult females whereas most other classes of genes were expressed in the antennae of both males and females, indicating their diversity in importance for the olfactory sensory system. Together, transcriptome profiling of *C. vestalis* genes involved in the antennal odorant-sensory system helps in detecting genes involved in host- and food-search behaviors through infochemically-mediated interactions.

## Introduction

In insect sensory systems antennae, maxillary palps and/or labial palps are the main olfactory organs. In these specific parts, olfactory sensory neurons (OSNs) are enclosed in hair-like cuticular structures with multiple pores, called sensilla. The OSN dendrites are submersed in the sensillum lymph that contains water soluble odorant binding proteins (OBPs) and chemosensory proteins (CSPs) [1]. Some enzymes, specifically expressed in the antennae [pheromone degrading enzymes (PDE) or carboxylesterase (CCE)] are also secreted and thought to be involved in signal inactivation [2], [3]. The two large gene families expressing OBPs and odorant receptors (ORs), however, characterize the molecular basis of insect olfaction and are assumed to be exclusive to this group of animals [4].

OBPs are small (10 to 30 kDa) globular and highly abundant proteins. They are thought to be involved in the uptake of volatile, hydrophobic compounds from the environment and their translocation to odorant receptors located in the ORN membranes [5]–[8]. Additionally, OBPs have been suggested to filter or purify odorants [8], [9], to act as activator factors of ORs (after conformational change) [10] or as carriers expressed in non-olfactory tissue [11], [12].

Insect ORs have recently been recognized as ligand-gated ion channels causing an inward cationic current [13], [14]. *In vivo* and *in vitro* assays show that ORs are active in a heteromeric complex of unknown stoichiometry composed of the OR and the universal co-receptor (ORCO) [14]–[17].The ORs are thought to be essential for the recognition of a variety of odorant compounds [18], [19] and to be involved in the pore construction and current determination [20], whereas ORCO is required for neuronal cell-surface targeting and proper signal transduction [21], [22].

Olfaction is important for fundamental behaviors such as feeding, mating and interaction with a prey or host. *Cotesia vestalis* (Haliday) (Hymenoptera: Braconidae) is an endoparasitic wasp that attacks larvae of the diamondback moth (DBM), *Plutella xylostella* L. (Lepidoptera: Yponomeutidae), an oligophagous herbivore of cruciferous plants. Female *C. vestalis* use herbivore-induced plant volatiles (HIPVs) from plants infested by *P. xylostella* larvae as host-searching cues [23]. A mixture of four HIPVs (*n*-heptanal, α-pinene, sabinene and (*Z*)-3-hexenyl acetate) released from *P. xylostella*-infested cabbage plants triggers innate chemotaxis in naive parasitoids, whereas the individual compounds by themselves do not [24]. Therefore, it is not a single HIPV but rather a blend of different HIPVs that is recognized by the natural enemies of herbivores [24]–[26]. However, little is known about the sensory mechanisms underlying complicated HIPV recognition.

We surveyed sensory genes, especially for ORs and OBPs, expressed in the antennae of *C. vestalis* adult females by Roche 454 transcriptome pyrosequencing to partially evaluate the odorant-sensory systems. To date, transcriptome pyrosequencing is the most widely used system for *de novo* sequencing and analysis of transcriptomes especially in non-model organisms. In addition, the OR and some of OBP genes identified were examined in order to evaluate their tissue and gender-specific patterns of expression.

**Table 1:** Summary of the materials and results of pyrosequencing.

Category	Library 1	Library 2	Library 3-1	Library 3-2	Total
RNA source	TotalRNA	mRNA	mRNA	mRNA	
cDNA amplification	NuGEN	NuGEN	Roche Std.	Roche Std.	
Run regions	1/8	1/8	1/8	1/8	
Valid reads	76 892	110 009	122 436	133 578	442 915
Valid reads(minus rRNA)	32 093	93 009	56 899	65 360	247 361
Number ofcontigs					17 328
Number ofsingletons					31 921
Average sizeof contigs					549
					

## Materials and Methods

### Plants and Insects

Cabbage plants (*Brassica oleracea* L. cv. Shikidori) were cultivated in a growth chamber at 25°C with a photoperiod of 16 h (natural+supplemental light). DBM (*P. xylostella* L. [Lepidoptera: Yponomeutidae]) were collected originally from fields of Kyoto Prefecture, Japan, in 2001. No specific permits were required for the field studies. The land was not privately owned or under any statutory protection and the field studies did not involve endangered or protected species. DBM collected were mass-reared on potted cabbage plants in a climate-controlled room (25°C, 16 h photoperiod). *C. vestalis* (Hymenoptera: Braconidae) were obtained from the above field-collected, parasitized larvae of DBM. To obtain further generations of *C. vestalis*, adult wasps were placed with DBM larvae on cabbage plants in plastic-glass cages (25×35×30 cm; three windows covered by nylon gauze and one door for introducing plants and wasps). These cages were maintained in a climate-controlled room (25°C, 16 h photoperiod) for 4 days. The parasitized DBM larvae were isolated and held in a plastic container (1.6 L) with a few cabbage leaves for a week. Every *C. vestalis* cocoon was transferred into a plastic cage (0.2 L) and incubated until the adults emerged (4–7 days). The emerged adult wasps were collected within 24 h and temporarily stored at −80°C. This amounted to 720 females and 680 males sampled wasps. Each part of *C. vestalis* (antenna, head and body) was dissected on dry ice and collected in an Eppendorf tube. The tubes contained either 60 antennae, 30 heads or 30antennae and each tube constituted a unit sample. All tubes were stored at −80°C until processed.

**Figure 1:**
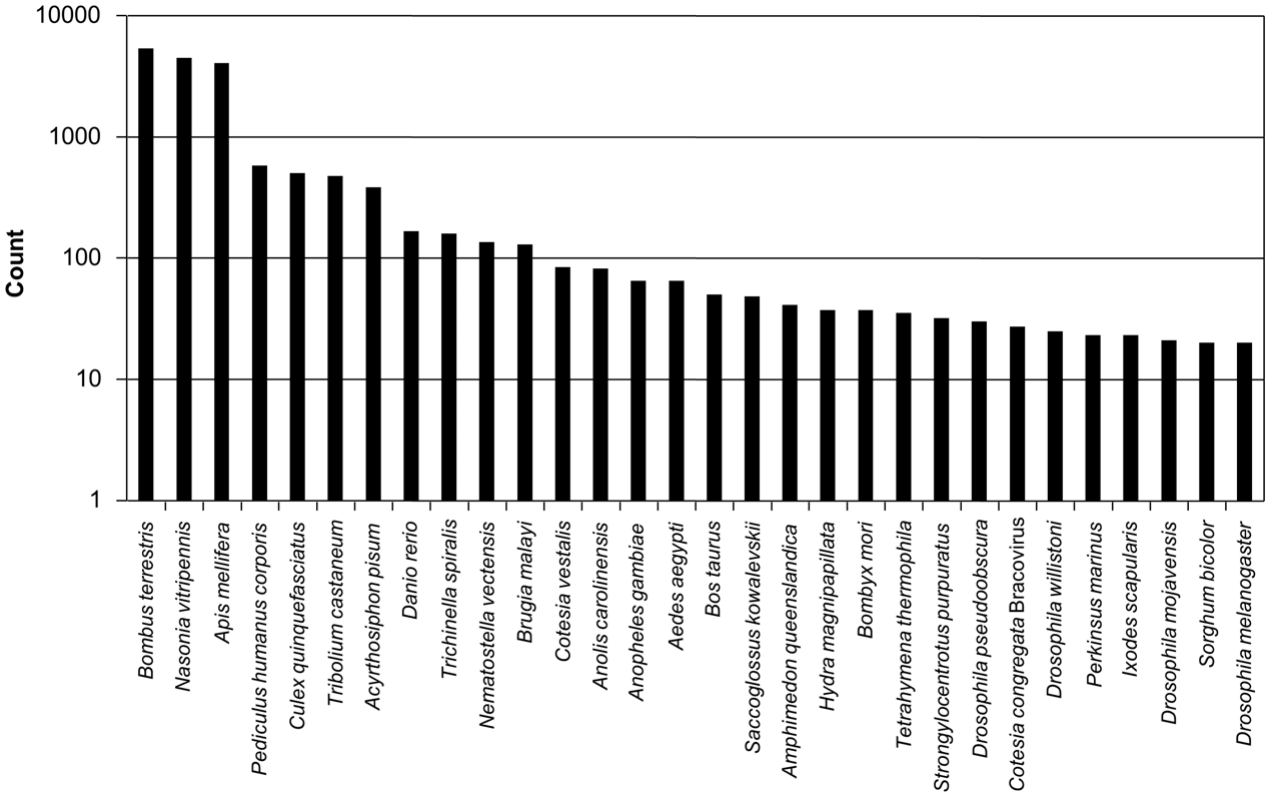
Species distribution of top hits for the pyrosequencing contigs/singletons from *C. vestalis* adult female antennae (*n* = 17 597; 35.7%, BLAST hits).

**Figure 2:**
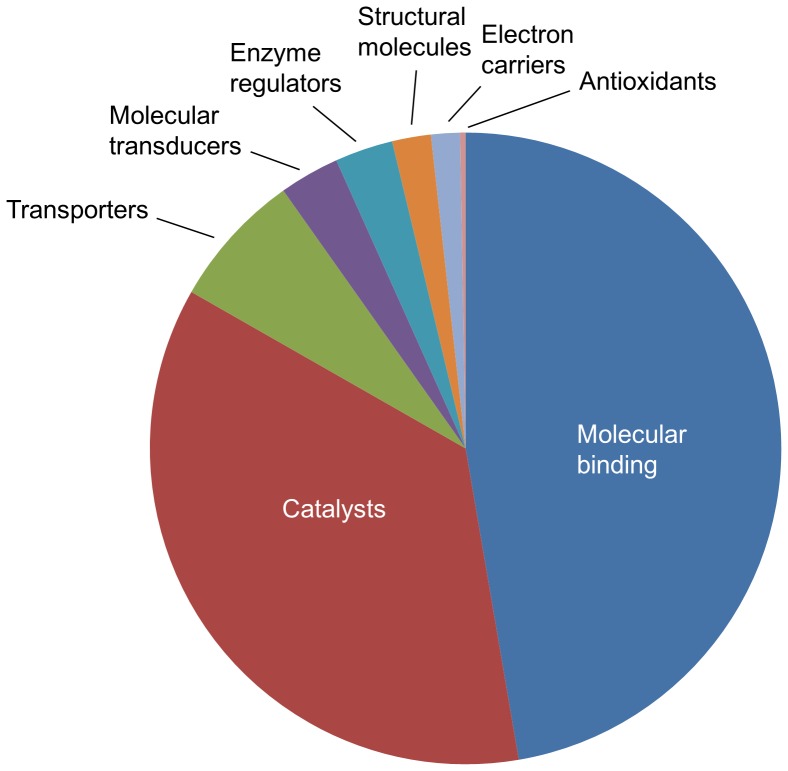
Distribution of transcriptome pyrosequencing from contigs/singletons. The major categories of level 2 molecular functions from a Gene Ontology (GO) analysis are shown (*n* = 10 642).

### RNA Preparation, cDNA Library Construction and 454 Sequencing

Total RNA from 6 unit samples (a total of 360 female antennae) was isolated using a Qiagen RNeasy Mini Kit and an RNase-Free DNase Set (Qiagen, Hilden, Germany) following the manufacturer’s protocol. Total RNA (4.5 µg) was then taken through mRNA purification using a RiboMinus Eukaryote Kit for RNA-Seq (Invitrogen, Carlsbad, CA, USA) according to the manufacturer’s instructions (yielding 600 ng mRNA). Total RNA and mRNA quality were assessed by an Agilent Bioanalyzer RNA 6000 Pico Assay (Agilent Technologies, Santa Clara, CA, USA) and quantified by a Quant-iT RiboGreen Assay (Invitrogen).

**Table 2:** The part of contigs from *Cotesia vestalis* with similarity to OR genes.

Name	Contig	Read count	Length (bp)	Homology	E-value*
OR1	Cv_002063	293	1999	Olfactory receptor [*Microplitis mediator*]	0
OR2	Cv_000919	24	842	Odorant receptor 44 [*Nasonia vitripennis*]	2E−44
OR3	Cv_000252	15	992	Putative odorant receptor 13a [*Harpegnathos saltator*]	7E−32
OR4	Cv_005941	14	688	Odorant receptor 265 [*N. vitripennis*]	7E−23
OR5	Cv_006402	10	1005	Odorant receptor 37 [*Tribolium castaneum*]	8E−15
OR6	Cv_006618	10	1236	Putative odorant receptor 13a [*H. saltator*]	3E−45

Contigs which are regenerated at least from 10 reads are listed. *We identified genes whose E-values exhibited at least 1E-5 or less, with the BLAST searches.

**Table 3:** The part of contigs from *Cotesia vestalis* with similarity to OBP genes.

Name	Contig	Read count	Length (bp)	Homology	E-value*
OBP1	Cv_001562	2184	1276	Odorant-binding protein 3 [*Microplitis mediator*]	5E−15
OBP2	Cv_002750	1998	1139	Pheromone-binding protein 1 [*M. mediator*]	4E−61
OBP3	Cv_000569	770	952	Odorant-binding protein 10 [*M. mediator*]	1E−65
OBP4	Cv_002480	430	1212	Odorant-binding protein 2 [*M. mediator*]	1E−57
OBP5	Cv_003043	399	1186	Odorant-binding protein 4 [*M. mediator*]	6E−34
OBP6	Cv_001669	230	1082	Odorant-binding protein 4 [*M. mediator*]	7E−23
OBP7	Cv_001286	162	1326	Odorant-binding protein 6 [*M. mediator*]	3E−61
OBP8	Cv_001766	123	1013	Odorant-binding protein 3 [*M. mediator*]	2E−28
OBP9	Cv_003903	74	642	Odorant-binding protein 3 [*M. mediator*]	1E−35
OBP10	Cv_001246	71	980	Odorant-binding protein 18 [*Nasonia vitripennis*]	9E−32
OBP11	Cv_000714	63	970	Odorant-binding protein 10 [*M. mediator*]	5E−62
OBP12	Cv_002207	41	1736	Odorant-binding protein 1 [*M. mediator*]	7E−54
OBP13	Cv_000843	27	594	Pheromone-binding protein 1 [*M. mediator*]	1E−16
OBP14	Cv_000016	26	590	Odorant-binding protein 10 [*M. mediator*]	4E−47
OBP15	Cv_000232	23	599	Pheromone-binding protein 1 [*M. mediator*]	8E−42
OBP16	Cv_001034	17	551	Odorant-binding protein 3 [*M. mediator*]	6E−10
OBP17	Cv_005637	16	804	Odorant-binding protein 6 [*M. mediator*]	3E−19
OBP18	Cv_005027	15	501	Odorant-binding protein 4 [*M. mediator*]	6E−8
OBP19	Cv_001050	14	714	Odorant-binding protein 3 [*M. mediator*]	1E−20
OBP20	Cv_006168	12	632	Odorant-binding protein 71 [*N. vitripennis*]	5E−7
OBP21	Cv_001120	12	896	Odorant-binding protein 2 [*M. mediator*]	6E−29
OBP22	Cv_001116	10	737	Odorant-binding protein 10 [*M. mediator*]	8E−23

Contigs which are regenerated at least from 10 reads are listed. *We identified genes whose E-values exhibited at least 1E-5 or less, with the BLAST searches.

Some of the odorant-sensory genes were predicted to be expressed at very low levels. In order to evenly mine genes expressed in the antenna at widely dissimilar levels, we prepared 3 libraries constructed using three types of cDNA sources: amplified cDNAs from total RNA (library 1), amplified cDNAs from mRNA (library 2), and non-amplified cDNAs from mRNA (library 3). The cDNAs were synthesized and amplified from mRNA or total RNA (50 ng each) with the Ovation RNA-Seq System (NuGEN, San Carlos, CA, USA) according to the manufacturer’s instructions, and the amplified double-stranded cDNA fragments were used in the following steps. For the preparation of non-amplified cDNAs, the mRNA (200 ng) was fragmented in 10 mM ZnCl_2_ and 10 mM Tris-HCl (pH 7.0) at 70°C for 30 s, and the double-stranded cDNA was synthesized using the cDNA Synthesis System Kit with random hexamer primers (Roche Applied Science, Indianapolis, IN, USA). The cDNA fragments were subjected to ligation to the sequencing adaptors provided with the GS FLX Titanium Rapid Library Preparation Kit (Roche Applied Science), and small fragments were removed with AMPure XP (Beckman Coulter, Fullerton, CA, USA). Sequencing was performed on a GS FLX platform with Titanium chemistry (Roche/454) using a Medium/Small region of a PicoTiterPlate (PTP) per library, following the manufacturer’s instructions. Libraries 1 and 2 were sequenced using one region on the plate and library 3 using two.

**Figure 3:**
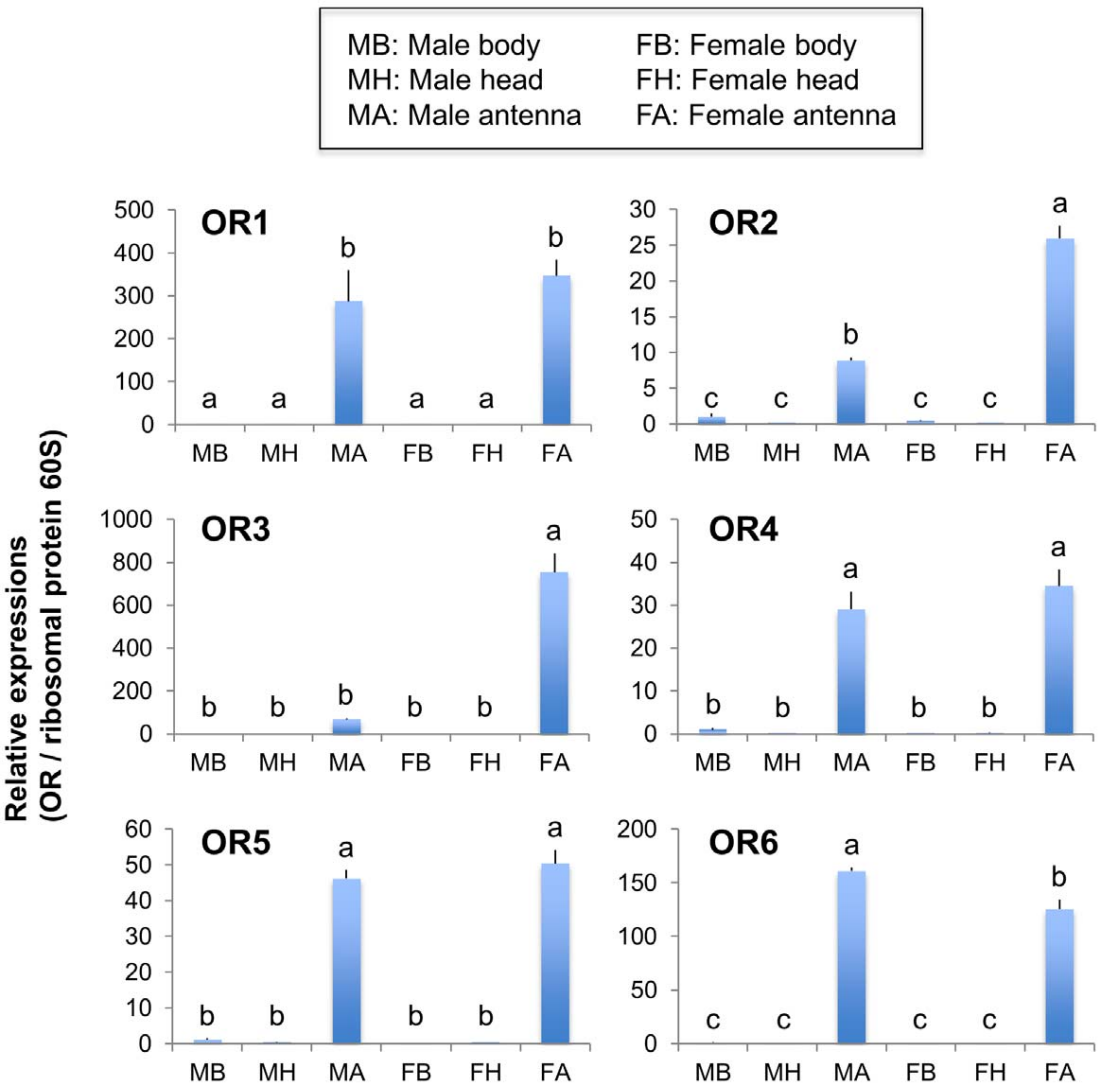
Tissue and genderspecific expressions of OR genes in *C. vestalis* adults. Transcription levels of genes for OR1 to 6 were normalized to those of *C. vestalis* 60S ribosomal protein L10 (Cv_000471), and expressed relative to the normalized transcript levels in the body of *C. vestalis* males. Data represent the mean and standard errors (*n = *5). Means followed by different small letters are significantly different (*P*<0.05, Tukey-Kramer HSD test).

**Figure 4:**
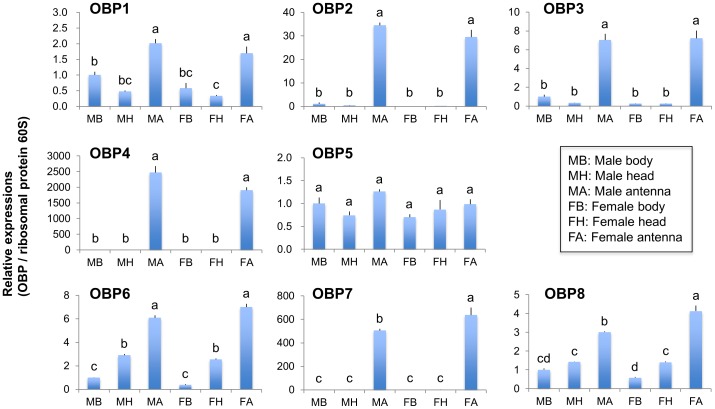
Tissue and genderspecific expressions of OBP genes in *C. vestalis* adults. Transcription levels of genes for OBP1 to 10 were normalized to those of *C. vestalis* 60S ribosomal protein L10 (Cv_000471), and expressed relative to the normalized transcript levels in the body of *C. vestalis* males. Data represent the mean and standard errors (*n = *5). Means followed by different small letters are significantly different (*P*<0.05, Tukey-Kramer HSD test).

### 454 *de novo* Transcriptome Assembly and Analysis

A pool of the processed sequence reads from the cDNA libraries was clustered and assembled using the TGICL pipeline version 2.1 [27] with a minimum sequence overlap of 49 nt and a minimum percentage overlap identity of 80%. The primary assembling eventually resulted in 17 328 contigs from the valid sequence reads. To annotate the dataset, both the contigs and singletons were searched for sequence similarity using BLASTX [28] against the NCBI RefSeq protein database (http://www.ncbi.nlm.nih.gov/RefSeq/), and the Gene Ontology (GO) analysis was conducted using the UniProt-GOA database (http://www.ebi.ac.uk/GOA/). In order to specifically annotate OBP and OR genes, the contigs and singletons were also searched for sequence similarity using BLASTX against the reported OBP and OR sequences of *Nasonia vitripennis* [29], [30] and the UniProtKB/TrEMBL database (http://www.ebi.ac.uk/uniprot/). Contigs and singletons with an E-value of 1E-5 or lower, on the BLAST hits, were used.

### Quantitative Reverse Transcriptase (RT)-PCR

Five independent replications of total RNA samples were prepared from about 30 body, 120 head or 480 antenna tissues of *C. vestalis* males or females, using a Qiagen RNeasy Mini Kit and an RNase-Free DNase Set (Qiagen) following the manufacturer’s protocol. First-strand cDNA was synthesized using a PrimeScript RT reagent Kit (Takara, Otsu, Japan), and 0.5 µg of total RNA at 37°C for 15 min. Real-time PCR was done on an Applied Biosystems 7300 Real-Time PCR System (Foster City, CA, USA) using Power SYBR Green Master Mix (Applied Biosystems), cDNA (1 µl from 20 µl of each RT product pool), and 300 nM primers. The PCR program was: initial polymerase activation 2 min at 50°C, and 10 min at 95°C, 40 cycles of 15 s at 95°C, and 60 s at 60°C. PCR conditions were chosen by comparing threshold values in a dilution series of the RT product, followed by non-RT template control and non-template control for each primer pair. Relative RNA levels were calibrated and normalized with the level of those of 60S ribosomal protein L10 (Cv_000471) for *C. vestalis*. Primers for real-time PCR were designed using the Primer-BLAST (http://blast.ncbi.nlm.nih.gov/Blast.cgi) for a length of the resulting PCR product of approximately 150 bp. Primers used for this study are shown in Table S1.

## Results

### An Overview of the Transcriptome of the Antennae of Adult *C. vestalis* Female

We constructed three libraries using three different types of cDNA sources: amplified cDNAs from total RNA (library 1), amplified cDNA from mRNA (library 2), and two non-amplified cDNAs from mRNA (libraries 3-1 and 3-2). From these the pyrosequencing yielded 76 892, 110 009, 122 436 and 133 578 valid reads, respectively, for a total of 442 915 reads (Table 1). However, after excluding ribosomal RNA (rRNA), the number of valid reads strongly decreased to 247 361 and showed that only library 2 was not contaminated with rRNA (93 009 valid reads minus rRNA). This was probably due to the enrichment of mRNA through two rounds of mRNA purification: the first using the RiboMinus Eukaryote Kit (Invitrogen) after purification; and the second using the Ovation RNA-Seq System (NuGEN), in which Oligo dT primers were used for RT reaction (see Materials and Methods). After cleaning the data, the high quality reads were assembled into 17 328 contigs and 31 921 singletons (Table 1; DDBJ accession number: DRA000551). The contigs had an average size of 549 base pairs (bp).

The BLAST searches of contigs/singletons against the UniProtKB/TrEMBL database found the *C. vestalis* sequences to be similar to amino acid sequences from two bee species *Apis mellifera* (4051 hits with E-values ≤1E-5) and *Bombus terrestris* (5346 hits) and the parasitoid wasp *Nasonia vitripennis* (4454 hits) (Figure 1). Sequences from some other insects such as *Pediculus humanus corporis*, *Culex quinquefasciatus*, *Tribolium castaneum*, and *Acyrthosiphon pisum* were also similar to *C. vestalis* contigs/singletons, whereas the model insect *Drosophila* sp. showed low-hit similarities.

Molecular function distributions from the Gene Ontology (GO) analyses showed that in *C. vestalis* the genes expressed in the antennae were mostly linked to molecular binding activity (e.g., nucleotide, ion and odorant binding; in 47% of the total scores) or categorized as catalysts (e.g., hydrolase and oxidoreductase; in 36% of the total scores) (Figure 2). The contigs/singletons involved in molecular transporters and transducers (including odorant receptors) constitute the next most abundant categories amounting to 7 and 3%, respectively.

### Annotation of a Part of OR and OBP Genes

Contigs and singletons with at least one match to ORs and OBPs and with an E-value of 1E-5 or lower were selectively annotated. BLAST search indicated that ORs were predicted in 64 contigs and 99 singletons, and that OBPs were predicted in 74 contigs and 71 singletons (Tables 2, 3, S2 and S3). The number of predicted OBP contigs regenerated from at least 10 reads were 22 (Table 3), but only six were regenerated from OR contigs (Table 2 **)**. Therefore, in the antennae of female *C. vestalis*, OBP genes appeared to be expressed more abundantly than OR genes. OBP1 and OBP2 in particular were expressed at remarkably high levels.

### Expression Patterns of *C. vestalis* OR and OBP Genes

We conducted quantitative RT-PCR analyses in different tissues (bodies, heads and antennae) of adult males and females to assess the expression of *C. vestalis* OR1 to 6 and OBP1 to 10. These contigs are regenerated from at least 100 and 10 sequence reads, respectively. Analyses showed that all the OR genes analyzed were expressed in an antenna-specific manner (Figure 3). The expression of genes for OR2 and OR3 in the antennae of female wasps was much higher than in the antennae of male wasps, while an OR6 gene was expressed at a slightly lower level in females than in males. OBP genes had three different expression patterns: i) ubiquitously expressed (OBP5), ii) barely antenna-specific (OBP1, 6 and 8), and iii) highly antenna-specific (OBP2, 3, 4 and 7) (Figure 4). Moreover, genes for OBP7 and 8 were expressed at slightly higher levels in the antennae of female wasps than in those of males.

## Discussion

Quality-checked transcriptome pyrosequencing reads of genes expressed in the antennae of *C. vestalis* adult females assembled into 17 328 contigs, including 64 OR and 74 OBP contigs. The parasitoid wasp, *Nasonia vitripennis*, which has similar genetic traits as *C. vestalis* (Figure 1), has a total of 301 OR genes, of which 225 are intact genes and 76 are pseudogenes [29]. Similarly, honey bee genome analyses revealed a major expansion of the OR family to 174 genes, encoding 163 potentially functional receptors, at least twice as many as those found in *Drosophila melanogaster* [29]. The total number of OR contigs and singletons found in the pyrosequences of *C. vestalis* antennal genes is 163 (Table S2). This large repertoire of OR genes might enable the wasp to sense the wide range of pheromones, floral scents, HIPVs and other olfactory cues. However, low expression levels of some OR genes prevented us from mining all members of this superfamily by transcriptomes. For a more comprehensive analysis, genomic analyses of *C. vestalis* should be conducted. In contrast, OBP genes were likely to be expressed at much higher levels in *C. vestalis* antennae, resulting in a total of 74 contigs and 71 singletons from large numbers of valid sequence reads (e.g., 2184 reads for OBP1 and 1998 reads for OBP2, Tables 3 and S3). The number of contigs obtained is comparable to that found in other insect species, such as the 66 putative OBP genes found in the mosquito *Aedes aegypti* [31]. While OR expression is generally restricted to neurons of the olfactory sensilla, OBP expression is associated with both olfactory and gustatory sensilla [32]. This may in part account for the different expression thresholds between OR and OBP genes.

There were trends in the antennal-specific transcription pattern of OR and OBP genes **(** Figures 3 and 4). Predominantly, OR2, OR3, OBP7 and OBP8 genes were expressed in female antennae. These genes could play a role in odorant perception of certain HIPVs or sex pheromone in the specific host-searching and oviposition behavior [23] of female *C. vestalis*. *Microplitis mediator* OBP6, the homologue of *C. vestalis* OBP7 (Figure S1), has been reported to bind specifically to several plant odorants [33]. For ORs, however, little is known about odorant ligands and most of the valid data have been obtained from *Drosophila* spp. [34], [35]. Female *C. vestalis* are attracted selectively to a specific blend of DBM-induced cabbage volatiles including (*Z*)-3-hexenol, *n*-heptanal, α-pinene, sabinene, (*Z*)-3-hexenyl acetate, limonene, (*E*)-4,8-dimethyl-1,3,7-nonatriene and camphor [24]. These host-searching cues can be specifically recognized by antennal-specific ORs and OBPs. However, there is little to suggest chemosensory difference between adult male and female antennae or between adult male and female maxillary palps in mosquitoes. Neither tissue shows significant sexual dimorphism with respect to the types of sensilla they bear [36] or stimuli they respond to [37]. If the same holds true for parasitoid wasps, all antennal-specific OBPs and ORs, expressed irrespective of their sex and not only by females, may function in odorant recognition. Otherwise, the interplay of several OBPs and ORs is likely to be important for the recognition of general odorants and specific HIPVs. Functional characterization of these odorant-sensory proteins, with the extensive cloning of some full-length cDNAs, and *in situ* localization analysis will help to understand *C. vestalis* chemoreception mechanisms.

## Supporting Information

Figure S1 **Phylogenetic alignment of the amino acid sequence of OBPs.** The OBPs derived from five different Hymenoptera species were aligned and organized in a phylogenic tree: *Microplitis mediator* (Mm); *Nasonia vitripennis* (Nv); *Vepsa crabro* (Vc), *Polistes dominulus* (Pd) and the current study [*Cotesia vestalis* (Cv)]. Alignments were conducted with ClustalW and MUCLE. Neighbor-joining tree was constructed using MEGA5 and presented with 50% cut-off bootstrap value of 1 000 bootstrap replicates sampled. The numbers at the branching points are bootstrap values (%). GenBank accession numbers of protein sequences are: MmOBP1, ABM05968; MmOBP2, ABM05969; MmOBP3, ABM05970; MmOBP4, ABM05971; MmOBP5, ABM05972; MmOBP6, ABO015559; MmOBP7, ABM05973; MmOBP10, AEO27860; MmCSP1, ABO15560; NvOBP, XP_001601068; NvOBP, XP_001603472.2; NvOBP71, XP_001601290; NvOBP70, CCD17839; NvOBP1, XP_001606873; DmOBP56e, ABW77913; PdOBP1, AAP55718; PdCSP1, AAP55719; VcCSP1, AAV68929.
(TIF)

Table S1 **RT-PCR primers used for this study.**
(XLS)

Table S2 **All the sets of contigs and singletons from** ***Cotesia vestalis*** **with similarity to OR genes.**
(XLS)

Table S3 **All the sets of contigs and singletons from** ***Cotesia vestalis*** **with similarity to OBP genes.**
(XLS)
